# Deregulation of sale of over-the-counter drugs outside of pharmacies in the Republic of Korea: interrupted-time-series analysis of outpatient visits before and after the policy

**DOI:** 10.1186/s12913-017-2434-6

**Published:** 2017-07-12

**Authors:** Sung-Youn Chun, Hye-Ki Park, Kyu-Tae Han, Woorim Kim, Hyo-Jung Lee, Eun-Cheol Park

**Affiliations:** 1 Department of Public Health, Graduate School, Yonsei University, Seoul, Republic of Korea; 2 Institute of Health Services Research, Yonsei University College of Medicine, 50 Yonsei-ro, Seodaemun-gu, Seoul, 120-752 Republic of Korea; 3 Department of Preventive Medicine, Yonsei University College of Medicine, 50 Yonsei-ro, Seodaemun-gu, Seoul, 120-752 Republic of Korea

**Keywords:** Pharmacy, Policy, Korea, Nonprescription drugs, Outpatients

## Abstract

**Background:**

We evaluated the effectiveness of a policy allowing for the sale of over-the-counter drugs outside of pharmacies by examining its effect on number of monthly outpatient visits for acute upper respiratory infections, dyspepsia, and migraine.

**Method:**

We used medical claims data extracted from the Korean National Health Insurance Cohort Database from 2009 to 2013. The Korean National Health Insurance Cohort Database comprises a nationally representative sample of claims - about 2% of the entire population - obtained from the medical record data held by the Korean National Health Insurance Corporation (which has data on the entire nation). The analysis included26,284,706 person-months of 1,042,728 individuals. An interrupted-time series analysis was performed. Outcome measures were monthly outpatient visits for acute upper respiratory infections, dyspepsia, and migraine. To investigate the effect of the policy, we compared the number of monthly visits before and after the policy’s implementation in 2012.

**Result:**

For acute upper respiratory infections, monthly outpatient visits showed a decreasing trend before the policy (ß = −0.0003);after it, a prompt change and increasing trend in monthly outpatient visits were observed, but these were non-significant. For dyspepsia, the trend was increasing before implementation (ß = −0.0101), but this reversed after implementation(ß = −0.007). For migraine, an increasing trend was observed before the policy (ß = 0.0057). After it, we observed a significant prompt change (ß = −0.0314) but no significant trend.

**Conclusion:**

Deregulation of selling over-the-counter medication outside of pharmacies reduced monthly outpatient visits for dyspepsia and migraine symptoms, but not acute upper respiratory infections.

**Electronic supplementary material:**

The online version of this article (doi:10.1186/s12913-017-2434-6) contains supplementary material, which is available to authorized users.

## Background

Before November 2012, it was forbidden in the Republic of Korea to sell medicine or medical supplies in places other than pharmacies. This included both prescribed pharmaceuticals and over-the-counter drugs. However, this meant that many people were unable to buy necessary or desired medication at night or during holidays, when pharmacies are closed. The social need for greater access to this medication eventually became too great to ignore, prompting the government to address the issue. As a result, in November 2012, the government implemented an over-the-counter drug policy that permitted the sale of over-the-counter drugs such as cold and digestive medicines, antipyretics, analgesics, and pain relief patches in places outside of the pharmacy [1].

In the United States of America, the United Kingdom, Germany, and numerous countries, over-the-counter drugs are legally sold in convenience stores and supermarkets [2]. As such, there have been many previous studies focusing on the myriad impacts of these drugs. For instance, Smith and Feldman performed a systematic review of clinical trials of over-the-counter drugs, and found that some over-the-counter drugs, whether on their own or in combination, have positive effects on reducing cold symptoms(i.e., upper respiratory tract infections)in adolescents and adults [3]. In contrast, Schroederand Fahey suggested that over-the-counter cough medicines were ineffective for reducing acute cough in adults [4]. Other than investigating the effectiveness of over-the-counter medications themselves, many studies have focused on the side effects of these drugs, which remain a major concern [5, 6]. Furthermore, children’s caregivers may lack sufficient knowledge of the appropriate dosages or types of over-the-counter drugs to utilize them safely [7], and many studies, including more recent ones, have reported notable rates of drug abuse and adverse effects related to using over-the-counter medications [8–11].

Despite the plethora of information on over-the-counter drugs, the actual effects of over-the-counter drug policies on the population remain unknown. Specifically, it remains unclear whether being able to access over-the-counter drugs at places outside the pharmacy has reduced medical service use and the number of outpatients for conditions such as acute upper respiratory infections; this effect is particularly difficult to evaluate because the sale of over-the-counter drugs at places outside the pharmacy has already been unregulated long ago in many countries [12].

Given this lack of information on the effectiveness of over-the-counter drug policies, we sought to evaluate the effectiveness of the policy implemented in the Republic of Korea in 2012 for reducing the number of outpatient visits for acute upper respiratory infections, dyspepsia, and migraine by comparing these numbers before and after its implementation.

## Methods

We used medical claims data extracted from the Korean National Health Insurance Cohort Database from 2009 to 2013. The Korean National Health Insurance Cohort Database comprises a nationally representative sample of claims—about 2% of the entire population—obtained from the medical record data held by the Korean National Health Insurance Corporation (which has data on the entire nation) [13]. The specific data used included details of each patient’s utilization of healthcare. We handled each patient’s medical record data by month in order to analyze patients’ trends over time. Overall, the analysis included 26,284,706 person-months from 1,042,728 individuals. This study was approved by the institutional review board at Yonsei University Graduate School of Public Health (2014–239). Because patients’ information was anonymized before the analysis, we did not require the informed consent of participants.

### Design

We used an interrupted time-series analysis to evaluate the trends over time in monthly outpatient visits in the Republic of Korea for the above three conditions [14–16]. Interrupted time-series is appropriate for investigation of intervention and it’s longitudinal effects over time. Our data composed by a series of measurements over time interrupted by an intervention. We performed segmented linear regression model with individual-level data which includes segments of pre-intervention and post-intervention [17, 18].

$$ {\mathrm{Y}}_{\mathrm{t}\kern0.5em }={\upbeta}_0+{\upbeta}_1\times {\mathrm{Time}}_{\mathrm{t}}+{\upbeta}_2\times {\mathrm{Intervention}}_{\mathrm{t}}+{\upbeta}_3\times {\mathrm{Time}\ \mathrm{after}\ \mathrm{intervention}}_{\mathrm{t}}+{\upbeta}_4\times {\mathrm{Covariates}}_{\mathrm{t}}\kern0.5em +{\mathrm{e}}_{\mathrm{t}} $$

Our main intervention is implementation of the policy permitting the sale of over-the-counter drug sat places outside the pharmacy.

### Outcome measures

Over-the-counter drug policy that permitted the sale of over-the-counter drugs was mainly about cold and digestive medicines, antipyretics. Therefore, outcome measures included the number of outpatient visits per month for complaints of acute upper respiratory infections, dyspepsia, and migraine which is target disease of permitted drugs. The following codes from the International Statistical Classification of Diseases and Related Health Problems, 10th Revision, were used to identify outpatient visits for these conditions: acute upper respiratory infections (J00 to J06), dyspepsia(K30,F45.3, and R12), and migraine(G43 and G44).

### Independent variables

The intervention variable was the implementation of the policy permitting the sale of over-the-counter drugs at places outside the pharmacy. Thus, an “intervention” effect refers to a prompt change in the number of monthly outpatient visits just after implementation of the over-the-counter drug policy in November 2012. The “trend before policy” refers to the trend in monthly outpatient visits before the implementation of the over-the-counter drug policy, while the “trend after policy” refers to the trend in monthly outpatient visits after implementation of the policy.

For covariates, we assessed the general characteristics of sex, age (20–40 years, 40–60 years, 60–80 years, or >80 years), type of insurance (employee insurance, self-employed insurance, or Medicaid), income (four quartiles), region (urban or rural), and type of hospital usually used for the outcome measure symptoms (general hospital, hospital, clinic, or others). Furthermore, we included the Elixhauser comorbidity index as a measure of medical condition. This index comprises 31 categories of comorbid diseases [19], and allowed us to control for certain critical medical conditions that would influence individuals’ well-being, such as peptic ulcer disease, paralysis, peripheral vascular disorders, valvular disease, neurological disorders and rheumatoid arthritis/collagen disorders, metastatic cancer, obesity, alcohol abuse, and drug abuse (see Additional file 1).

### Statistical analysis

We aggregated patients’ medical records by month so that each patient had a maximum of 48 repeated measures (i.e., 4 years). We used t-tests to identify differences in the mean number monthly outpatient visits by patients’ characteristics. We used a generalized estimating equation model to account for the longitudinal nature of the data and thereby ensure an appropriate analysis. Furthermore, we used an autoregressive covariance structure and a negative binomial distribution to account for the interrupted time series design and distribution of monthly outpatient visits in our data [20, 21]. All analyses were performed using the SAS software package (ver. 9.3; SAS Institute, Inc., Cary, NC).

## Results

Study participants’ general characteristics are listed in Table 1. Of the 10,42,718patients, 49.8% were male and 39.24% were under 20 years old. Furthermore, 46.8% of patients were living in urban areas. Most patients 59.5% usually visited clinics for acute upper respiratory infections; 77% did so for dyspepsia and migraine.

**Table 1:** Patients’ general characteristics

		Number	Percent
Sex			
Male		518,793	49.8
Female		523,935	50.3
Age			
less than 30		409,186	39.2
30–39		165,379	15.9
40–49		177,031	17.0
50–59		135,173	13.0
60–69		84,577	8.1
70–79		52,063	5.0
80 or more		19,319	1.9
Insurance			
Self-employed insured		353,906	33.9
employee insured		655,998	62.9
Medicaid		32,824	3.2
Income			
Q1(Low)		167,277	16.0
Q2		248,196	23.8
Q3		342,296	32.8
Q4(High)		284,959	27.3
Region			
Urban		487,892	46.8
Rural		554,836	53.2
Type of hospital (Acute upper respiratory infection)			
General hospital		100,539	9.6
Hospital		92,976	8.9
Clinic		620,058	59.5
Others		229,155	22.0
Type of hospital (Dyspepsia)			
General hospital		107,329	10.3
Hospital		105,076	10.1
Clinic		803,233	77.0
Others		27,090	2.6
Type of hospital (Migraine)			
General hospital		106,703	10.2
Hospital		104,797	10.1
Clinic		802,351	77.0
Others		28,877	2.8
Total		1,042,728	100

Table 2 presents the mean monthly outpatient visits by outcome measure. For acute upper respiratory infections, the mean number of monthly outpatient visits was 0.2742. Female and younger patients tended to have more outpatient visits than did male and older ones, respectively (Male: 0.2880; Female: 0.3137), (Less than 30: 0.4031; 30–39: 0.2895; 40–49: 0.2351; 50–59: 0.2125; 60–69: 0.2055; 70–79: 0.2049; more than 80: 0.1321). Patients who has high income tended to have more outpatient visits than who has low income (1st Quartile: 0.2734; 2nd Quartile: 0.2858; 3rd Quartile: 0.3204; 4th Quartile: 0.3068). Korea mainly have three types of health insurance (Self-employed insured, employee insured, Medicaid). Patients who have employee insured tended to have more outpatient visits than patients who have other insurance (Self-employed insured: 0.2881;employee insured: 0.3109; Medicaid: 0.2392).

**Table 2:** Mean number of monthly outpatient visits for acute upper respiratory infections, dyspepsia, and migraine

	Acute upper respiratory infections			Dyspepsia			Migraine		
	Mean	SD	*p*-value	Mean	SD	*p*-value	Mean	SD	*p*-value
Sex									
Male	0.2880	0.6873	<.0001	0.0036	0.0718	<.0001	0.0046	0.0814	<.0001
Female	0.3137	0.7211		0.0041	0.0781		0.0075	0.1102	
Age									
less than 30	0.4031	0.7902	<.0001	0.0038	0.0702	<.0001	0.0034	0.0659	<.0001
30–39	0.2895	0.6551		0.0035	0.0746		0.0069	0.1032	
40–49	0.2351	0.6147		0.0035	0.0688		0.0083	0.1130	
50–59	0.2125	0.6260		0.0038	0.0812		0.0078	0.1087	
60–69	0.2055	0.6376		0.0042	0.0780		0.0073	0.1090	
70–79	0.2049	0.6808		0.0056	0.0900		0.0104	0.1519	
80 or more	0.1321	0.5058		0.0047	0.1179		0.0060	0.1021	
Insurance									
Self-employed insured	0.2881	0.6903	<.0001	0.0037	0.0729	0.0002	0.0063	0.0964	<.0001
employee insured	0.3109	0.7141		0.0039	0.0763		0.0058	0.0949	
Medicaid	0.2392	0.6593		0.0041	0.0714		0.0094	0.1358	
Income									
Q1(Low)	0.2734	0.6754	<.0001	0.0037	0.0730	<.0001	0.0071	0.1058	<.0001
Q2	0.2858	0.6810		0.0039	0.0785		0.0059	0.0993	
Q3	0.3204	0.7249		0.0039	0.0735		0.0054	0.0860	
Q4(High)	0.3068	0.7160		0.0038	0.0749		0.0062	0.1019	
Region									
Urban	0.2993	0.7104	0.0007	0.0036	0.0735	<.0001	0.0061	0.1004	<.0001
Rural	0.3023	0.6995		0.0040	0.0763		0.0060	0.0939	
Type of hospital									
General hospital	0.1063	0.4213	<.0001	0.0031	0.0598	<.0001	0.0099	0.1058	<.0001
Hospital	0.1999	0.5582		0.0027	0.0551		0.0064	0.0931	
Clinic	0.3375	0.7456		0.0044	0.0828		0.0054	0.0961	
Others	0.3778	0.6915		0.0032	0.0655		0.0073	0.1020	
Total	0.2742	0.7321		0.0045	0.0870		0.0078	0.1171	

For symptoms of dyspepsia, the mean number of monthly outpatient visits was fewer than acute upper respiratory infections, which was 0.0045. As with acute upper respiratory infections, female patients tended to have greater mean number of visits for dyspepsia, while patients who did not have a comorbid disease tended to have a greater number of visits than did those who did have a comorbid disease(Male: 0.0036; Female: 0.0041). Older patients tended to have more outpatient visits than did younger ones(Less than 30: 0.0038; 30–39: 0.0035; 40–49: 0.0035; 50–59: 0.0038; 60–69: 0.0042; 70–79: 0.0056; more than 80: 0.0047). Patients who have Medicaid insurance tended to have more outpatient visits than patients who have other insurance (Self-employed insured: 0.0037; employee insured: 0.0039;Medicaid: 0.0041). For migraine, the mean number of monthly outpatient visits was0.0078. Older patients tended to have more outpatient visits than did younger ones, patients who have Medicaid insurance tended to have more outpatient visits than patients who have other insurance (Less than 30: 0.0034; 30–39: 0.0069; 40–49: 0.0083; 50–59: 0.0078; 60–69: 0.0073; 70–79: 0.0104; more than 80: 0.0060), (Self-employed insured: 0.0063; employee insured: 0.0058; Medicaid: 0.0094).

Table 3 presents the interrupted time-series analysis of the effect of the over-the-counter medication policy. Each number in Table 3 present change of monthly outpatient visits for three separate symptoms. For acute upper respiratory infections, we observed a significant decreasing trend in monthly outpatient visits before the policy implementation (ß = −0.0003). After implementing the policy, we observed a prompt change in monthly outpatient visits (ß = 0.0026) but this was not statistically significant. Similarly, the trend after the policy was now increasing, but not to a significant degree. In summary, the monthly outpatient visits for acute upper respiratory infections showed a decreasing trend before the policy, and implementing the policy had no significant effect.

**Table 3:** Interrupted time series analysis of the effect of the over-the-counter drug policy on number of monthly outpatient visits for acute upper respiratory infections, dyspepsia, and migraine

	Acute upper respiratory infections			Dyspepsia			Migraine		
	Estimation	Standard error	*p*-value	Estimation	Standard error	*p*-value	Estimation	Standard error	*p*-value
Trend	−0.0003*	0.0001	<.0001	0.0101*	0.0005	<.0001	0.0057*	0.0003	<.0001
Intervention									
Yes	0.0026	0.0017	0.1307	−0.0216	0.0194	0.2651	−0.0314*	0.0151	0.037
No	Ref.	-	-	Ref.	-	-	Ref.	-	-
Trend after intervention	0.0002	0.0002	0.3416	−0.007*	0.0021	0.0007	0.0019	0.0016	0.2497
Sex									
Male	−0.0137*	0.0015	<.0001	−0.2174*	0.0144	<.0001	−0.4796*	0.0149	<.0001
Female	Ref.	-	-	Ref.	-	-	Ref.	-	-
Age									
less than 30	0.568*	0.0107	<.0001	1.2492*	0.0406	<.0001	1.0934*	0.0429	<.0001
30–39	0.4174*	0.0108	<.0001	1.1252*	0.0422	<.0001	1.4585*	0.0426	<.0001
40–49	0.3975*	0.0108	<.0001	0.8341*	0.0426	<.0001	1.344*	0.0427	<.0001
50–59	0.3842*	0.0109	<.0001	0.462*	0.0433	<.0001	1.0162*	0.0429	<.0001
60–69	0.3177*	0.0113	<.0001	0.0865*	0.0438	0.0484	0.5039*	0.0441	<.0001
70–79	0.1888*	0.0115	<.0001	−0.0287	0.0448	0.5216	0.1723*	0.0451	0.0001
80 or more	Ref.	-	-	Ref.	-	-	Ref.	-	-
Insurance									
Self-employed insured	Ref.	-	-	Ref.	-	-	Ref.	-	-
employee insured	0.0004	0.0062	0.9423	0.0868*	0.0424	0.0406	0.1812*	0.0406	<.0001
Medicaid	0.0215*	0.0063	0.0006	0.1346*	0.0438	0.0021	0.1915*	0.042	<.0001
Income									
Q1(Low)	−0.0459*	0.0024	<.0001	−0.2681*	0.0225	<.0001	−0.1883*	0.0198	<.0001
Q2	−0.0407*	0.002	<.0001	−0.2768*	0.0183	<.0001	−0.2068*	0.017	<.0001
Q3	0.0047*	0.0016	0.0043	−0.185*	0.0167	<.0001	−0.1625*	0.0154	<.0001
Q4(High)	Ref.	-	-	Ref.	-	-	Ref.	-	-
Region									
Urban	0.0646*	0.0015	<.0001	0.5416*	0.0137	<.0001	0.5124*	0.013	<.0001
Rural	Ref.	-	-	Ref.	-	-	Ref.	-	-
Type of hospital									
General hospital	−0.0509*	0.0071	<.0001	−0.0013*	0.0385	<.0001	−0.0149*	0.0275	<.0001
Hospital	−0.0132*	0.0047	<.0001	−0.0062*	0.0284	<.0001	−0.058*	0.0208	<.0001
Clinic	−0.002*	0.0044	<.0001	0.1791*	0.0245	<.0001	−0.0505*	0.0184	<.0001
Others	Ref.	-	-	Ref.	-	-	Ref.	-	-
Admission in last month									
Yes	Ref.	-	-	Ref.	-	-	Ref.	-	-
No	0.0262*	0.0031	<.0001	−0.2025*	0.0257	<.0001	−0.1626*	0.0197	<.0001
Season									
Spring	0.0113*	0.0009	<.0001	0.0046	0.0102	0.654	0.0762*	0.0075	<.0001
Summer	−0.0801*	0.0011	<.0001	0.0147	0.011	0.181	0.136*	0.008	<.0001
Fall	−0.0065*	0.0009	<.0001	−0.0495*	0.0106	<.0001	0.0512*	0.0076	<.0001
Winter	Ref.	-	-	Ref.	-	-	Ref.	-	-

*: *p*-value < 0.05; All covariates are adjusted

For symptoms of dyspepsia, a significant increasing trend before the policy was observed(ß = −0.0101). While implementing the policy did not induce a significant prompt reduction in monthly outpatient visits, the decreasing trend after the policy was significant (ß = −0.007). Thus, the over-the-counter drug policy may have changed the trend in outpatient visits for dyspepsia.

For migraine, the trend before the policy was increasing (ß = 0.0057). After the policy implementation, we observed a prompt decrease in monthly outpatient visits (ß = −0.0314). However, there was no significant trend.

Tables 4 and 5 presents subgroup analysis of interrupted time-series by income. Table 4 shows effect of the over-the-counter medication policy on low-income population, and Table 5 shows effect of the over-the-counter medication policy on high-income population. Total trend of the monthly outpatient visits in all symptoms were similar in both sub-population and statistically significant. In acute upper respiratory infections, there were prompt increase in monthly outpatient visits among low-income population after the intervention, but trend after intervention was decreasing and it was not statistically significant (Intervention: 0.0052; trend after intervention: −0.0002). In high-income population, prompt increase after intervention were smaller than low-income population, but trend after intervention was increasing but it was not statistically significant (Intervention: 0.0014; trend after intervention: 0.0004). In dyspepsia, monthly outpatient visits were decreased after the intervention in both sub-population, but only trend after intervention were statistically significant (Trend after intervention: −0.0066(low-income), −0.0078(high-income)). For migraine, there were prompt decrease in low-income population after the intervention and it was statistically significant (Intervention: −0.0801). Trend after intervention was increasing but it was not statistically significant. In high-income population, nothing was statistically significant.

**Table 4:** Interrupted time series analysis of the effect of the over-the-counter drug policy on number of monthly outpatient visits for acute upper respiratory infections, dyspepsia, and migraine (Low-income patients)

	Acute upper respiratory infections			Dyspepsia			Migraine		
	Estimation	Standard error	*p*-value	Estimation	Standard error	*p*-value	Estimation	Standard error	*p*-value
Trend	−0.0003*	0.0001	<.0001	0.0102*	0.0008	<.0001	0.0067*	0.0006	<.0001
Intervention									
Yes	0.0052	0.003	0.0854	−0.0488	0.0307	0.1114	−0.0801*	0.0241	0.0009
No	Ref.	-	-	Ref.	-	-	Ref.	-	-
Trend after intervention	−0.0002	0.0003	0.5126	−0.0066*	0.0032	0.0398	0.0039	0.0025	0.1256

*: *p*-value < 0.05; All covariates are adjusted

**Table 5:** Interrupted time series analysis of the effect of the over-the-counter drug policy on number of monthly outpatient visits for acute upper respiratory infections, dyspepsia, and migraine (High-income patients)

	Acute upper respiratory infections			Dyspepsia			Migraine		
	Estimation	Standard error	*p*-value	Estimation	Standard error	*p*-value	Estimation	Standard error	*p*-value
Trend	−0.0003*	0.0001	<.0001	0.0102*	0.0006	<.0001	0.0053*	0.0004	<.0001
Intervention									
Yes	0.0014	0.0021	0.4997	−0.0083	0.0248	0.7362	0.001	0.0199	0.9614
No	Ref.	-	-	Ref.	-	-	Ref.	-	-
Trend after intervention	0.0004	0.0002	0.0824	−0.0078*	0.0027	0.0036	−0.0005	0.0023	0.8152

*: *p*-value < 0.05; All covariates are adjusted

**Fig. 1:**
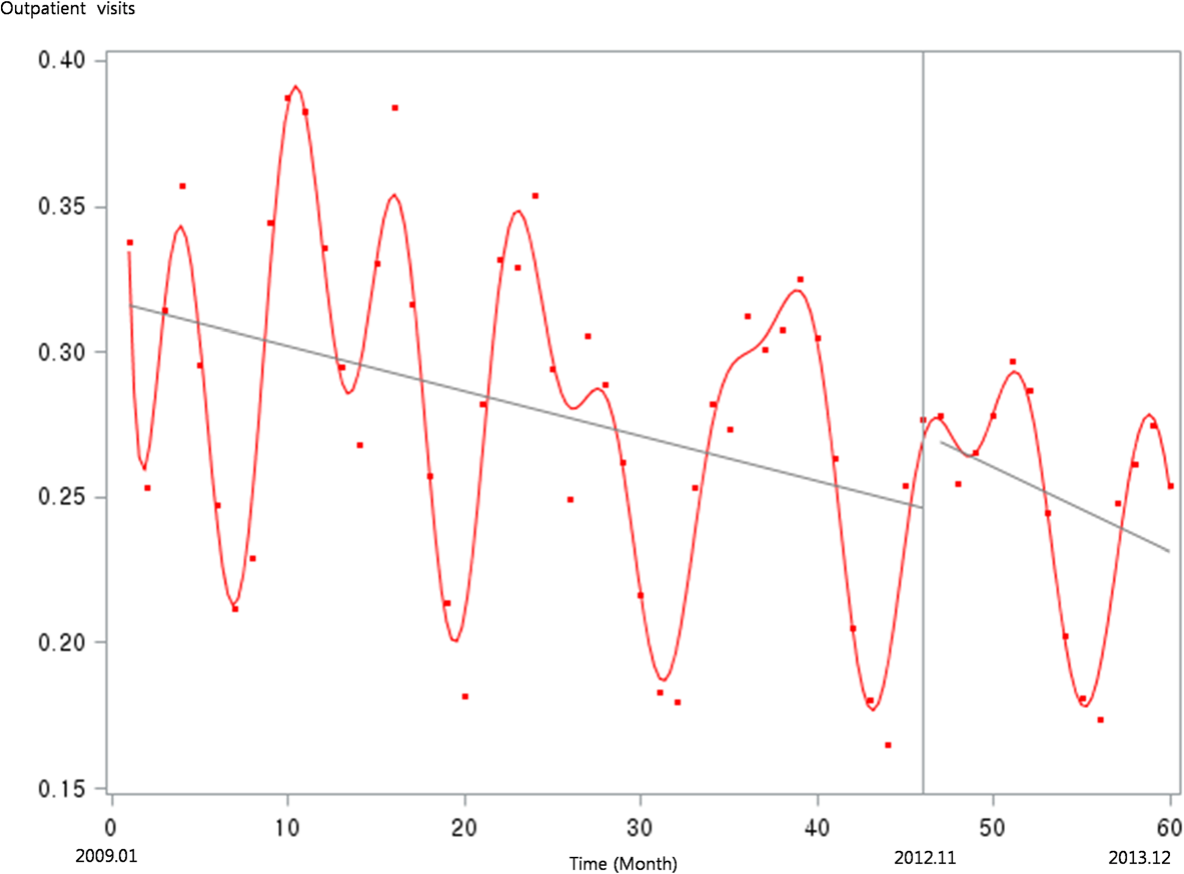
Unadjusted graph of outpatient visits for acute upper respiratory infections

**Fig. 2:**
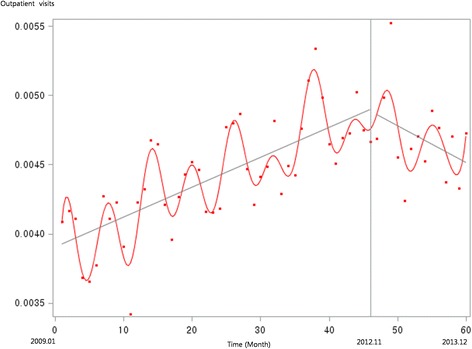
Unadjusted graph of outpatient visits for dyspepsia symptoms

**Fig. 3:**
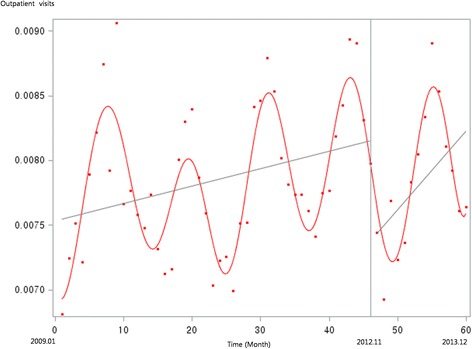
Unadjusted graph of outpatient visits for migraine

**Fig. 4:**
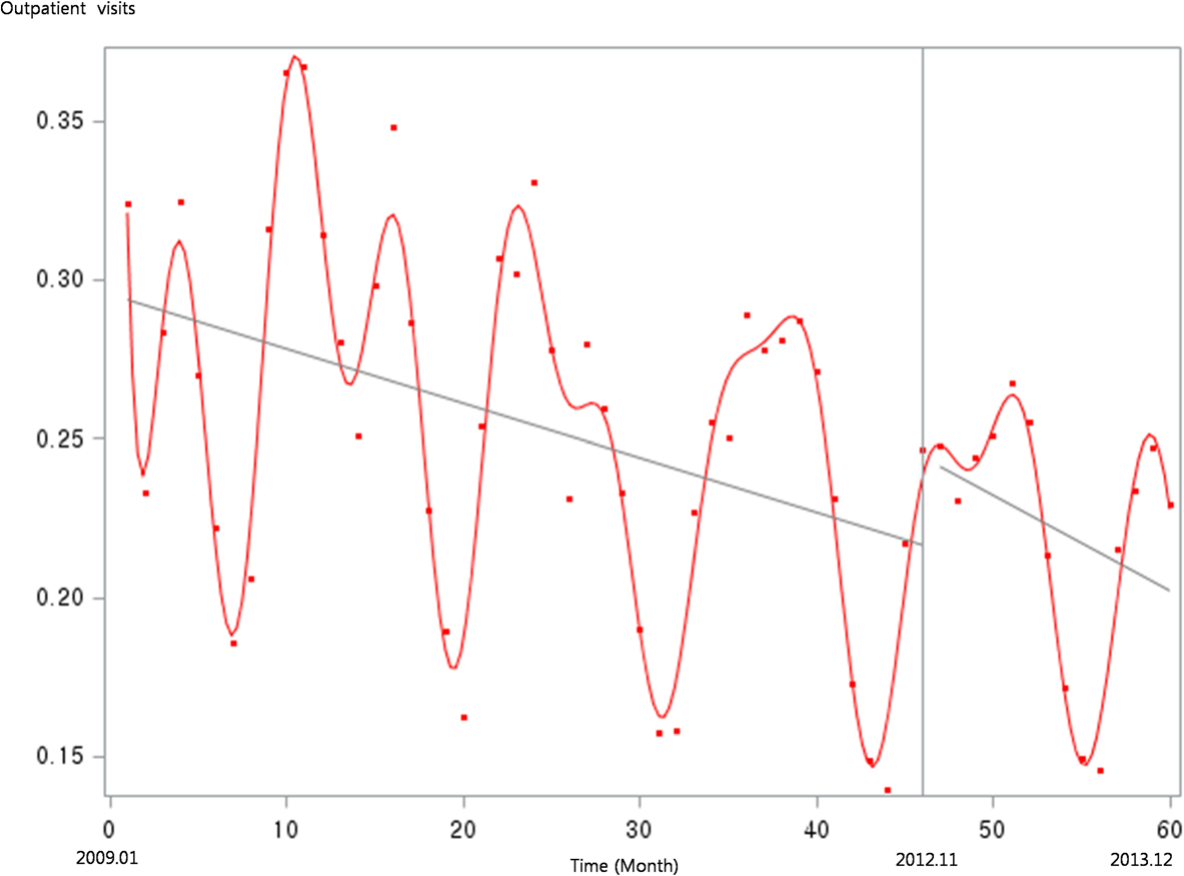
Unadjusted graph of outpatient visits for acute upper respiratory infections (low-income patients)

**Fig. 5:**
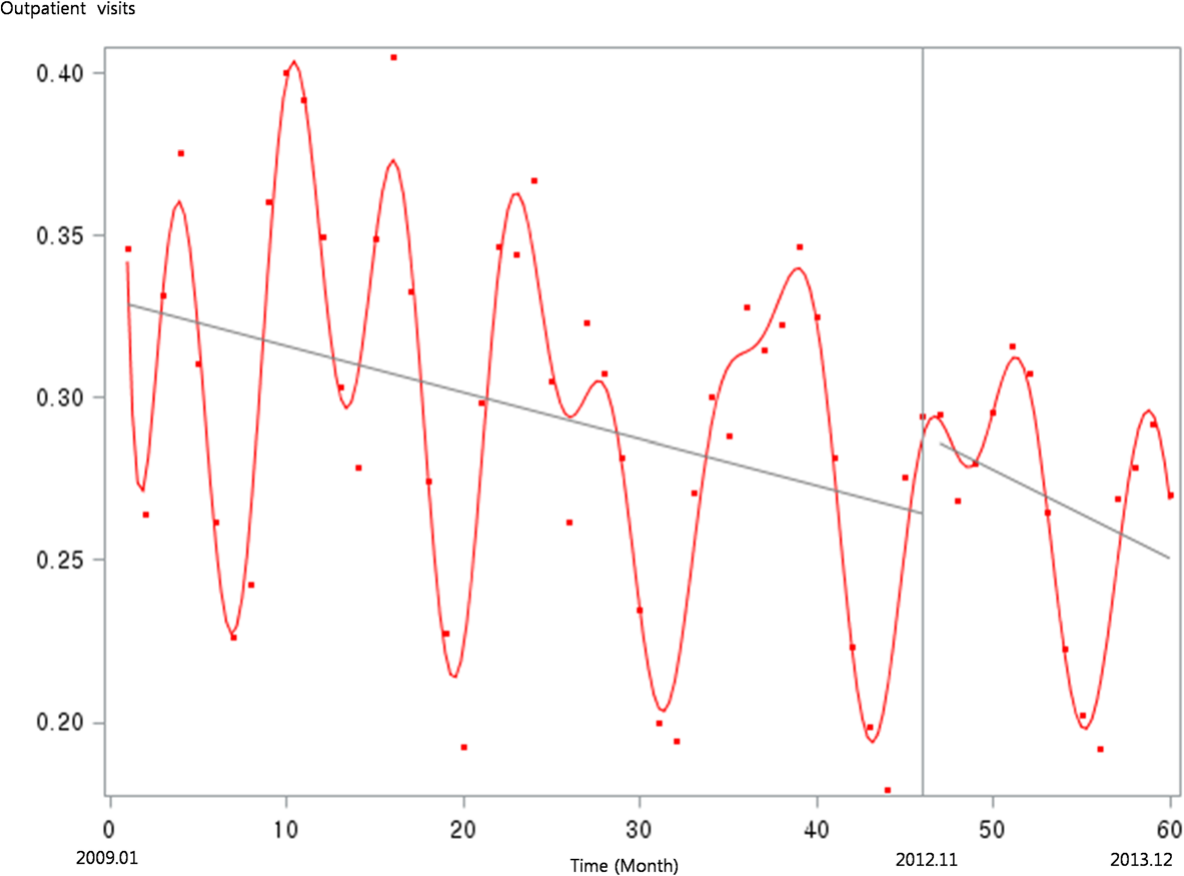
Unadjusted graph of outpatient visits for acute upper respiratory infections (high-income patients)

**Fig. 6:**
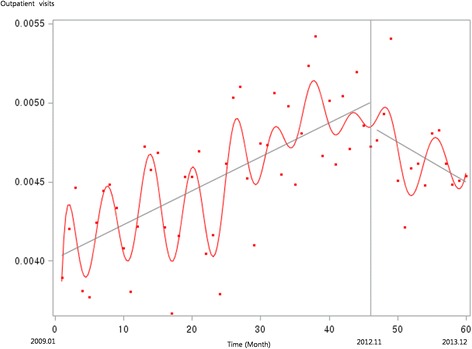
Unadjusted graph of outpatient visits for dyspepsia symptoms (low-income patients)

**Fig. 7:**
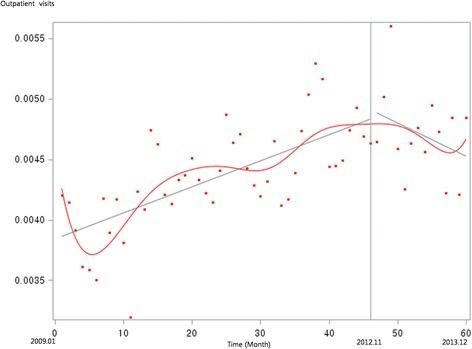
Unadjusted graph of outpatient visits for dyspepsia symptoms (high-income patients)

**Fig. 8:**
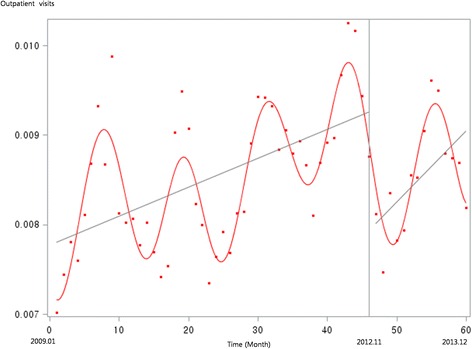
Unadjusted graph of outpatient visits for migraine (low-income patients)

**Fig. 9:**
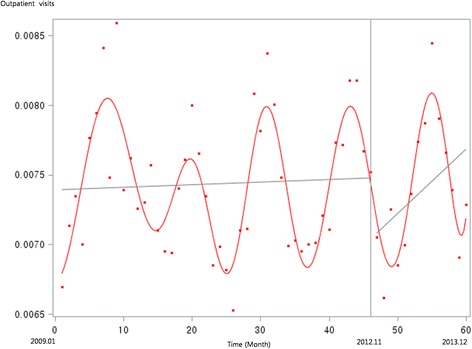
Unadjusted graph of outpatient visits for migraine (high-income patients)

Figure 1 presents the unadjusted graph of outpatient visits for acute upper respiratory infections. Notably, the number of visits showed a decreasing trend before the intervention, but implementation of the policy led to a prompt increase in the number of visits. Figure 2 presents the unadjusted graph of outpatient visits for dyspepsia symptoms. Before the intervention, the number of outpatient visits showed an increasing trend. However, it began to noticeably decrease after implementation of the over-the-counter medication policy. Finally, Fig. 3 presents the number of outpatient visits for migraine. Although implementing the policy appears to have led to a prompt decrease in the number of visits, this number there after appeared to increase somewhat sharply. Figures 4, 5, 6, 7, 8, 9 presents the number of outpatient visits for acute upper respiratory infections, dyspepsia, and migraine among sub-population divided by income. Prompt effect of the policy was different between high-income population and low-income population, but the trend after intervention in prompt change after the policy, but the slope after intervention were similar in all sub-population.

## Discussion

Utility is a general concept for measuring the value that individuals attach to the outcomes of various courses of action [22]. The population of the Republic of Korea demanded medical accessibility, and the government agreed that such accessibility had more value than others. As result, the policy permitting the sale of over-the-counter drugs at places outside the pharmacy was implemented. However, we must consider the tradeoffs of implementing this policy.

As noted in the Introduction, over-the-counter drugs have been studied in some detail. Qato et al. showed how, in the United States, there was a slight decrease in the prevalence of using over-the-counter drugsbetween2005 and2011;even so, over 35% of the entire nation’s population still used these drugs in 2011 [23]. The high usage of over-the-counter drugs has led to identification of the myriad side effects of such drugs. For instance, Kogan et al. found that53.7% of sampled 3-year-old children had been given over-the-counter drugs that they should not have in the past 30 days [24]. Aside from the problem of misusing over-the-counter drugs, there is the problem of their being abused. According to Wazaify et al., “misuse” is defined as using an over-the-counter drug for an appropriate medical reason but in wrong doses or for a longer period than recommended [25]. In contrast, “abuse” is defined as using an over-the-counter drug for non-medical reasons such as losing weight or getting high. In Wazaify et al., almost 85% of participants thought that over-the-counter drugs could be abused.

Despite the potential adverse effects, misuse, and abuse of over-the-counter drugs, a policy permitting the sale such drugs at places outside of pharmacies would enable greater accessibility to medical treatment for low-severity diseases or symptoms. The goal of the present study was to evaluate the effect of this accessibility on outpatient visits. We found that acute upper respiratory infections had the most outpatient visits of any of the other conditions assessed. However, the over-the-counter drug policy did not significantly influence the number of visits for acute upper respiratory infections, and although the trend in outpatient visits tended to increase after implementing the policy, it was to a non-significant degree. One possible reason for this is that, although accessibility to drugs had increased after implementing the policy, the quality of medical services may have decreased because of the lack of prescriptions or physicians to provide advice on appropriate doses and recommended lengths of use, and to check patients’ status.

In contrast, the numbers of outpatient visits for symptoms of dyspepsia and migraine were influenced by the over-the-counter drug policy. As shown in Fig. 2, the slope of monthly outpatient visits for dyspepsia indicated an increasing trend before implementing the policy, after which it changed to a decreasing slope; the results of the interrupted time series analysis revealed the same trend. For symptoms of migraine, the number of monthly outpatient visits promptly decreased after implementing the policy, while the trend showed no significant difference between before and after the policy.

Overall, our findings suggest that the number of outpatient visits for symptoms of dyspepsia and migraine were influenced by the over-the-counter drug policy, while the number of visits for acute upper respiratory infections was not. However, the number of outpatient visits is but one of many ways to measure the effectiveness of the over-the-counter drug policy. As such, our results provide rather limited information on the overall effectiveness of the policy. For the same reason, although the over-the-counter drug policy did not have a dramatic effect on the number of outpatient visits, it would be unwise to conclude that it is not effective. We must consider the many other ways of measuring the effectiveness of the over-the-counter drug policy, especially changes in patients’ quality of life. We might, for instance, consider the sleep quality of people suffering from the pain of dyspepsia and migraine but who choose not to go to the hospital.

Bradley et al. suggested that a future trend for medication could be “self-care.” Once a physician has determined whether long-term or recurrent treatment is required, the treatment could be performed using over-the-counter medications at patients’ request [26]. However, to make such self-care work and to minimize the adverse effects, misuse, and abuse of over-the-counter drugs, several factors must be kept under control. First, deregulation of over-the-counter medication should be done gradually and carefully. In the Republic of Korea, there are 13 products of 4 types of medicine that have been designated as over-the-counter drugs. Radical deregulations must be avoided to prevent an increased incidence of side effects of over-the-counter drugs. Second, education and labeling of over-the-counter drugs must be done properly. According to Degrootet al.’s study, mandatory labeling requirements have reduced the prevalence of misuse of over-the-counter cough and cold medicine among children 1–5 years old from 22.2% to 17.8% [27]. Thus, we might prevent misuse of drugs for children just by labeling them properly. Finally, collaborative care is required between doctors, pharmacists, and patients themselves [26]. Patients must know what type and dose of over-the-counter drugs they must take, which would require suitable education from doctors and pharmacists.

There are some limitations to our study. First, as noted above, this study is on how the policy influence the outpatient number only, and no other impacts of the policy is studied. Further studies are required such as whether this policy induce more drug abuse and whether this policy reduce prevalence rate of target diseases. Also, there could be other factors influencing outpatient visits and interact with the implementation of the policy that we could not exclude. These confounding factors may invalidate the attribution of changes in outpatient visits to the policy. Second, we only have data until 2013, so we could not determine the long-term effects of the over-the-counter drug policy. One year after-policy period is not long enough. Further studies are needed after more data has been collected. Third, we did not include any socioeconomic variables other than income, which could lead to a bias in our results. Finally, we did not have any information about the severity of acute upper respiratory infections, dyspepsia, or migraine, which could have influenced the number of outpatient visits and therefore lead to bias.

Nevertheless, our study possesses a number of strengths. First, to our knowledge, it is the first to investigate the actual effect of the Republic of Korea’s over-the-counter drug policy on the population using an interrupted time series analysis. Second, because our data were sampled from the medical record data of the entire nation, our dataset can be nationally representative. Finally, we used a 5-year longitudinal dataset, therefore allowing for a highly accurate analysis.

## Conclusion

The deregulation of the over-the-counter drug policy reduced the number of monthly outpatient visits for dyspepsia and migraine, but not for acute upper respiratory infections. To ensure that the over-the-counter drug policy is effective, further studies are needed and deregulation should be performed slowly and carefully. Furthermore, patient education and drug labeling should be done properly, and collaboration between physicians, pharmacists, and patients is needed.

## Additional file

Additional file 1:
**Table S1.** Patients’ general characteristics (full model). **Table S2.** Mean number of monthly outpatient visits for acute upper respiratory infections, dyspepsia, and migraine (full model). **Table S3.** Interrupted time series analysis of the effect of the over-the-counter drug policy on number of monthly outpatient visits for acute upper respiratory infections, dyspepsia, and migraine (full model). (DOCX 80 kb)

## Abbreviations

AIDS: Acquired Immune Deficiency Syndrome
HIV: Human Immunodeficiency Virus
