# Supplementary material for: Deregulation of sale of over-the-counter drugs outside of pharmacies in the Republic of Korea: interrupted-time-series analysis of outpatient visits before and after the policy
Source: BMC Health Serv Res. 2017 Jul 12;17:478. doi: 10.1186/s12913-017-2434-6 (PMC5506678; doi:10.1186/s12913-017-2434-6)
Supplement: Additional file 1: — Table S1. Patients’ general characteristics (full model). Table S2. Mean number of monthly outpatient visits for acute upper respiratory infections, dyspepsia, and migraine (full model). Table S3. Interrupted time series analysis of the effect of the over-the-counter drug policy on number of monthly outpatient visits for acute upper respiratory infections, dyspepsia, and migraine (full model). (DOCX 80 kb) [file 12913_2017_2434_MOESM1_ESM.docx]

| **Table S1. Patients’ general characteristics (Full model)** | | | |
| --- | --- | --- | --- |
|  |  | **N** | **%** |
| **Sex** |  |  |  |
|  | Male | 518793 | 49.8 |
|  | Female | 523935 | 50.3 |
| **Age** |  |  |  |
|  | less than 30 | 409186 | 39.2 |
|  | 30-39 | 165379 | 15.9 |
|  | 40-49 | 177031 | 17.0 |
|  | 50-59 | 135173 | 13.0 |
|  | 60-69 | 84577 | 8.1 |
|  | 70-79 | 52063 | 5.0 |
|  | 80 or more | 19319 | 1.9 |
| **Insurance** |  |  |  |
|  | Self-employed insured | 353906 | 33.9 |
|  | employee insured | 655998 | 62.9 |
|  | Medicaid | 32824 | 3.2 |
| **Income** |  |  |  |
|  | Q1(Low) | 167277 | 16.0 |
|  | Q2 | 248196 | 23.8 |
|  | Q3 | 342296 | 32.8 |
|  | Q4(High) | 284959 | 27.3 |
| **Region** |  |  |  |
|  | Urban | 487892 | 46.8 |
|  | Rural | 554836 | 53.2 |
| **Type of hospital (Acute upper respiratory infection)** | |  |  |
|  | General hospital | 100539 | 9.6 |
|  | Hospital | 92976 | 8.9 |
|  | Clinic | 620058 | 59.5 |
|  | Others | 229155 | 22.0 |
| **Type of hospital (Dyspepsia)** | |  |  |
|  | General hospital | 107329 | 10.3 |
|  | Hospital | 105076 | 10.1 |
|  | Clinic | 803233 | 77.0 |
|  | Others | 27090 | 2.6 |
| **Type of hospital (Migraine)** | |  |  |
|  | General hospital | 106703 | 10.2 |
|  | Hospital | 104797 | 10.1 |
|  | Clinic | 802351 | 77.0 |
|  | Others | 28877 | 2.8 |
| **Congestive Heart Failure** | |  |  |
|  | No | 1038835 | 99.6 |
|  | Yes | 3893 | 0.4 |
| **Cardiac Arrhythmia** | |  |  |
|  | No | 1039461 | 99.7 |
|  | Yes | 3267 | 0.3 |
| **Valvular Disease** | |  |  |
|  | No | 1041992 | 99.9 |
|  | Yes | 736 | 0.1 |
| **Pulmonary Circulation Disorders** | |  |  |
|  | No | 1042592 | 100.0 |
|  | Yes | 136 | 0.0 |
| **Peripheral Vascular Disorders** | |  |  |
|  | No | 1030243 | 98.8 |
|  | Yes | 12485 | 1.2 |
| **Hypertension Uncomplicated** | |  |  |
|  | No | 970621 | 93.1 |
|  | Yes | 72107 | 6.9 |
| **Hypertension Complicated** | |  |  |
|  | No | 1036266 | 99.4 |
|  | Yes | 6462 | 0.6 |
| **Paralysis** |  |  |  |
|  | No | 1041152 | 99.9 |
|  | Yes | 1576 | 0.2 |
| **Other Neurological Disorders** | |  |  |
|  | No | 1037960 | 99.5 |
|  | Yes | 4768 | 0.5 |
| **Chronic Pulmonary Disease** | |  |  |
|  | No | 994998 | 95.4 |
|  | Yes | 47730 | 4.6 |
| **Diabetes Uncomplicated** | |  |  |
|  | No | 1024052 | 98.2 |
|  | Yes | 18676 | 1.8 |
| **Diabetes Complicated** | |  |  |
|  | No | 1029161 | 98.7 |
|  | Yes | 13567 | 1.3 |
| **Hypothyroidism** | |  |  |
|  | No | 1038904 | 99.6 |
|  | Yes | 3824 | 0.4 |
| **Renal Failure** | |  |  |
|  | No | 1041043 | 99.8 |
|  | Yes | 1685 | 0.2 |
| **Liver Disease** | |  |  |
|  | No | 1021994 | 98.0 |
|  | Yes | 20734 | 2.0 |
| **Peptic Ulcer Disease excluding bleeding** | | |  |
|  | No | 1018828 | 97.7 |
|  | Yes | 23900 | 2.3 |
| **AIDS/HIV** | |  |  |
|  | No | 1042673 | 100.0 |
|  | Yes | 55 | 0.0 |
| **Lymphoma** | |  |  |
|  | No | 1042568 | 100.0 |
|  | Yes | 160 | 0.0 |
| **Metastatic Cancer** | |  |  |
|  | No | 1042164 | 100.0 |
|  | Yes | 564 | 0.1 |
| **Solid Tumor without Metastasis** | |  |  |
|  | No | 1036722 | 99.4 |
|  | Yes | 6006 | 0.6 |
| **Rheumatoid Arthritis/collagen** | |  |  |
|  | No | 1038655 | 99.6 |
|  | Yes | 4073 | 0.4 |
| **Coagulopathy** | |  |  |
|  | No | 1042261 | 100.0 |
|  | Yes | 467 | 0.0 |
| **Obesity** |  |  |  |
|  | No | 1042517 | 100.0 |
|  | Yes | 211 | 0.0 |
| **Weight Loss** | |  |  |
|  | No | 1042300 | 100.0 |
|  | Yes | 428 | 0.0 |
| **Fluid and Electrolyte Disorders** | |  |  |
|  | No | 1039917 | 99.7 |
|  | Yes | 2811 | 0.3 |
| **Blood Loss Anemia** | |  |  |
|  | No | 1042412 | 100.0 |
|  | Yes | 316 | 0.0 |
| **Deficiency Anemia** | |  |  |
|  | No | 1038193 | 99.6 |
|  | Yes | 4535 | 0.4 |
| **Alcohol Abuse** | |  |  |
|  | No | 1039483 | 99.7 |
|  | Yes | 3245 | 0.3 |
| **Drug Abuse** | |  |  |
|  | No | 1042706 | 100.0 |
|  | Yes | 22 | 0.0 |
| **Psychoses** | |  |  |
|  | No | 1039624 | 99.7 |
|  | Yes | 3104 | 0.3 |
| **Depression** | |  |  |
|  | No | 1033417 | 99.1 |
|  | Yes | 9311 | 0.9 |
| **Total** |  | 1042728 | 100 |

| **Table S2. Mean number of monthly outpatient visits for acute upper respiratory infections, dyspepsia, and migraine (Full model)** | | | | | | | | | | | |
| --- | --- | --- | --- | --- | --- | --- | --- | --- | --- | --- | --- |
|  |  | **Acute upper respiratory infections** | | | | **Dyspepsia** | | | **Migraine** | | |
|  |  | **Mean** | | **SD** | **p-value** | **Mean** | **SD** | **p-value** | **Mean** | **SD** | **p-value** |
| **Sex** |  |  | |  |  |  |  |  |  |  |  |
|  | Male | 0.2880 | | 0.6873 | <.0001 | 0.0036 | 0.0718 | <.0001 | 0.0046 | 0.0814 | <.0001 |
|  | Female | 0.3137 | | 0.7211 |  | 0.0041 | 0.0781 |  | 0.0075 | 0.1102 |  |
| **Age** |  |  | |  |  |  |  |  |  |  |  |
|  | less than 30 | 0.4031 | | 0.7902 | <.0001 | 0.0038 | 0.0702 | <.0001 | 0.0034 | 0.0659 | <.0001 |
|  | 30-39 | 0.2895 | | 0.6551 |  | 0.0035 | 0.0746 |  | 0.0069 | 0.1032 |  |
|  | 40-49 | 0.2351 | | 0.6147 |  | 0.0035 | 0.0688 |  | 0.0083 | 0.1130 |  |
|  | 50-59 | 0.2125 | | 0.6260 |  | 0.0038 | 0.0812 |  | 0.0078 | 0.1087 |  |
|  | 60-69 | 0.2055 | | 0.6376 |  | 0.0042 | 0.0780 |  | 0.0073 | 0.1090 |  |
|  | 70-79 | 0.2049 | | 0.6808 |  | 0.0056 | 0.0900 |  | 0.0104 | 0.1519 |  |
|  | 80 or more | 0.1321 | | 0.5058 |  | 0.0047 | 0.1179 |  | 0.0060 | 0.1021 |  |
| **Insurance** |  |  | |  |  |  |  |  |  |  |  |
|  | Self-employed insured | 0.2881 | | 0.6903 | <.0001 | 0.0037 | 0.0729 | 0.0002 | 0.0063 | 0.0964 | <.0001 |
|  | employee insured | 0.3109 | | 0.7141 |  | 0.0039 | 0.0763 |  | 0.0058 | 0.0949 |  |
|  | Medicaid | 0.2392 | | 0.6593 |  | 0.0041 | 0.0714 |  | 0.0094 | 0.1358 |  |
| **Income** |  |  | |  |  |  |  |  |  |  |  |
|  | Q1(Low) | 0.2734 | | 0.6754 | <.0001 | 0.0037 | 0.0730 | <.0001 | 0.0071 | 0.1058 | <.0001 |
|  | Q2 | 0.2858 | | 0.6810 |  | 0.0039 | 0.0785 |  | 0.0059 | 0.0993 |  |
|  | Q3 | 0.3204 | | 0.7249 |  | 0.0039 | 0.0735 |  | 0.0054 | 0.0860 |  |
|  | Q4(High) | 0.3068 | | 0.7160 |  | 0.0038 | 0.0749 |  | 0.0062 | 0.1019 |  |
| **Region** |  |  | |  |  |  |  |  |  |  |  |
|  | Urban | 0.2993 | | 0.7104 | 0.0007 | 0.0036 | 0.0735 | <.0001 | 0.0061 | 0.1004 | <.0001 |
|  | Rural | 0.3023 | | 0.6995 |  | 0.0040 | 0.0763 |  | 0.0060 | 0.0939 |  |
| **Type of hospital** | |  | |  |  |  |  |  |  |  |  |
|  | General hospital | 0.1063 | | 0.4213 | <.0001 | 0.0031 | 0.0598 | <.0001 | 0.0099 | 0.1058 | <.0001 |
|  | Hospital | 0.1999 | | 0.5582 |  | 0.0027 | 0.0551 |  | 0.0064 | 0.0931 |  |
|  | Clinic | 0.3375 | | 0.7456 |  | 0.0044 | 0.0828 |  | 0.0054 | 0.0961 |  |
|  | Others | 0.3778 | | 0.6915 |  | 0.0032 | 0.0655 |  | 0.0073 | 0.1020 |  |
| **Congestive Heart Failure** | |  | |  |  |  |  |  |  |  |  |
|  | No | 0.3018 | | 0.7054 | 0.7905 | 0.0039 | 0.0751 | 0.0141 | 0.0061 | 0.0971 | 0.0251 |
|  | Yes | 0.0634 | | 0.3575 |  | 0.0013 | 0.0358 |  | 0.0015 | 0.0392 |  |
| **Cardiac Arrhythmia** | |  | |  |  |  |  |  |  |  |  |
|  | No | 0.3016 | | 0.7051 | 0.05 | 0.0039 | 0.0751 | 0.0021 | 0.0060 | 0.0970 | 0.0164 |
|  | Yes | 0.0790 | | 0.4655 |  | 0.0006 | 0.0247 |  | 0.0089 | 0.1031 |  |
| **Valvular Disease** | |  | |  |  |  |  |  |  |  |  |
|  | No | 0.3011 | | 0.7048 | 0.6724 | 0.0039 | 0.0750 | 0.7798 | 0.0060 | 0.0970 | 0.9772 |
|  | Yes | 0.0435 | | 0.2872 |  | 0.0014 | 0.0369 |  | 0.0014 | 0.0369 |  |
| **Pulmonary Circulation Disorders** | |  | |  |  |  |  |  |  |  |  |
|  | No | 0.3009 | | 0.7046 | 0.948 | 0.0038 | 0.0750 | 0.9821 | 0.0060 | 0.0970 | 0.9602 |
|  | Yes | 0.1250 | | 0.4625 |  | 0.0000 | 0.0000 |  | 0.0000 | 0.0000 |  |
| **Peripheral Vascular Disorders** | |  | |  |  |  |  |  |  |  |  |
|  | No | 0.3036 | | 0.7070 | 0.5766 | 0.0039 | 0.0751 | 0.0001 | 0.0060 | 0.0964 | <.0001 |
|  | Yes | 0.0783 | | 0.4125 |  | 0.0023 | 0.0675 |  | 0.0116 | 0.1367 |  |
| **Hypertension Uncomplicated** | |  | |  |  |  |  |  |  |  |  |
|  | No | 0.3179 | | 0.7199 | <.0001 | 0.0040 | 0.0757 | <.0001 | 0.0062 | 0.0982 | <.0001 |
|  | Yes | 0.0724 | | 0.3842 |  | 0.0018 | 0.0647 |  | 0.0041 | 0.0782 |  |
| **Hypertension Complicated** | |  | |  |  |  |  |  |  |  |  |
|  | No | 0.3024 | | 0.7058 | 0.02 | 0.0039 | 0.0752 | 0.0003 | 0.0061 | 0.0972 | <.0001 |
|  | Yes | 0.0684 | | 0.4050 |  | 0.0009 | 0.0305 |  | 0.0022 | 0.0465 |  |
| **Paralysis** |  |  | |  |  |  |  |  |  |  |  |
|  | No | 0.3013 | | 0.7050 | 0.3456 | 0.0039 | 0.0751 | 0.0252 | 0.0061 | 0.0970 | 0.35 |
|  | Yes | 0.0355 | | 0.2873 |  | 0.0019 | 0.0563 |  | 0.0013 | 0.0356 |  |
| **Other Neurological Disorders** | |  | |  |  |  |  |  |  |  |  |
|  | No | 0.3020 | | 0.7056 | 0.7228 | 0.0039 | 0.0752 | 0.3595 | 0.0060 | 0.0969 | 0.5923 |
|  | Yes | 0.0709 | | 0.3658 |  | 0.0015 | 0.0383 |  | 0.0103 | 0.1049 |  |
| **Chronic Pulmonary Disease** | |  | |  |  |  |  |  |  |  |  |
|  | No | 0.2993 | | 0.6998 | <.0001 | 0.0039 | 0.0751 | 0.143 | 0.0062 | 0.0983 | 0.0039 |
|  | Yes | 0.3339 | | 0.7977 |  | 0.0025 | 0.0742 |  | 0.0026 | 0.0619 |  |
| **Diabetes Uncomplicated** | |  | |  |  |  |  |  |  |  |  |
|  | No | 0.3051 | | 0.7084 | <.0001 | 0.0039 | 0.0753 | <.0001 | 0.0061 | 0.0971 | 0.0002 |
|  | Yes | 0.0733 | | 0.3897 |  | 0.0018 | 0.0562 |  | 0.0038 | 0.0904 |  |
| **Diabetes Complicated** | |  | |  |  |  |  |  |  |  |  |
|  | No | 0.3040 | | 0.7073 | 0.9555 | 0.0039 | 0.0754 | 0.3834 | 0.0061 | 0.0974 | 0.615 |
|  | Yes | 0.0662 | | 0.3963 |  | 0.0011 | 0.0374 |  | 0.0027 | 0.0582 |  |
| **Hypothyroidism** | |  | |  |  |  |  |  |  |  |  |
|  | No | 0.3017 | | 0.7054 | 0.7208 | 0.0039 | 0.0751 | 0.4156 | 0.0060 | 0.0970 | 0.6374 |
|  | Yes | 0.0766 | | 0.3863 |  | 0.0016 | 0.0396 |  | 0.0055 | 0.0839 |  |
| **Renal Failure** | |  | |  |  |  |  |  |  |  |  |
|  | No | 0.3014 | | 0.7050 | 0.6533 | 0.0039 | 0.0751 | 0.6849 | 0.0061 | 0.0971 | 0.9692 |
|  | Yes | 0.0332 | | 0.2288 |  | 0.0006 | 0.0244 |  | 0.0006 | 0.0244 |  |
| **Liver Disease** | |  | |  |  |  |  |  |  |  |  |
|  | No | 0.3055 | | 0.7090 | 0.5696 | 0.0039 | 0.0754 | 0.3402 | 0.0061 | 0.0971 | 0.1651 |
|  | Yes | 0.0743 | | 0.3736 |  | 0.0024 | 0.0542 |  | 0.0049 | 0.0930 |  |
| **Peptic Ulcer Disease excluding bleeding** | | | |  |  |  |  |  |  |  |  |
|  | No | | 0.3043 | 0.7075 | 0.5067 | 0.0038 | 0.0742 | <.0001 | 0.0060 | 0.0964 | <.0001 |
|  | Yes | | 0.1562 | 0.5498 |  | 0.0069 | 0.1031 |  | 0.0077 | 0.1187 |  |
| **AIDS/HIV** | | |  |  |  |  |  |  |  |  |  |
|  | No | | 0.3009 | 0.7046 | 0.5301 | 0.0038 | 0.0750 | 0.3368 | 0.0060 | 0.0970 | 0.3924 |
|  | Yes | | 0.0909 | 0.3482 |  | 0.0182 | 0.1348 |  | 0.0000 | 0.0000 |  |
| **Lymphoma** | | |  |  |  |  |  |  |  |  |  |
|  | No | | 0.3010 | 0.7047 | 0.5129 | 0.0038 | 0.0750 | 0.6764 | 0.0060 | 0.0970 | 0.623 |
|  | Yes | | 0.0375 | 0.2211 |  | 0.0000 | 0.0000 |  | 0.0000 | 0.0000 |  |
| **Metastatic Cancer** | | |  |  |  |  |  |  |  |  |  |
|  | No | | 0.3011 | 0.7047 | 0.95 | 0.0038 | 0.0750 | 0.754 | 0.0060 | 0.0970 | 0.704 |
|  | Yes | | 0.0337 | 0.3381 |  | 0.0035 | 0.0595 |  | 0.0018 | 0.0421 |  |
| **Solid Tumor without Metastasis** | | |  |  |  |  |  |  |  |  |  |
|  | No | | 0.3023 | 0.7060 | 0.0027 | 0.0039 | 0.0750 | 0.1145 | 0.0061 | 0.0971 | 0.0092 |
|  | Yes | | 0.0539 | 0.3271 |  | 0.0030 | 0.0729 |  | 0.0028 | 0.0805 |  |
| **Rheumatoid Arthritis/collagen** | | |  |  |  |  |  |  |  |  |  |
|  | No | | 0.3017 | 0.7053 | 0.2155 | 0.0039 | 0.0748 | 0.7353 | 0.0061 | 0.0971 | 0.0175 |
|  | Yes | | 0.1021 | 0.4448 |  | 0.0029 | 0.1151 |  | 0.0032 | 0.0564 |  |
| **Coagulopathy** | | |  |  |  |  |  |  |  |  |  |
|  | No | | 0.3010 | 0.7047 | 0.1848 | 0.0038 | 0.0750 | 0.5753 | 0.0060 | 0.0970 | 0.0564 |
|  | Yes | | 0.0428 | 0.2746 |  | 0.0043 | 0.0654 |  | 0.0000 | 0.0000 |  |
| **Obesity** |  | |  |  |  |  |  |  |  |  |  |
|  | No | | 0.3010 | 0.7047 | 0.6547 | 0.0038 | 0.0750 | 0.3104 | 0.0060 | 0.0970 | 0.5685 |
|  | Yes | | 0.1090 | 0.4603 |  | 0.0095 | 0.0971 |  | 0.0000 | 0.0000 |  |
| **Weight Loss** | | |  |  |  |  |  |  |  |  |  |
|  | No | | 0.3010 | 0.7047 | 0.5085 | 0.0038 | 0.0750 | 0.1982 | 0.0060 | 0.0970 | 0.2911 |
|  | Yes | | 0.0794 | 0.4871 |  | 0.0070 | 0.0835 |  | 0.0093 | 0.0963 |  |
| **Fluid and Electrolyte Disorders** | | |  |  |  |  |  |  |  |  |  |
|  | No | | 0.3011 | 0.7047 | 0.0007 | 0.0039 | 0.0751 | 0.2731 | 0.0060 | 0.0970 | 0.0093 |
|  | Yes | | 0.2398 | 0.6622 |  | 0.0028 | 0.0533 |  | 0.0071 | 0.1065 |  |
| **Blood Loss Anemia** | | |  |  |  |  |  |  |  |  |  |
|  | No | | 0.3010 | 0.7047 | 0.4503 | 0.0038 | 0.0750 | 0.9333 | 0.0060 | 0.0970 | 0.9522 |
|  | Yes | | 0.0823 | 0.5035 |  | 0.0032 | 0.0563 |  | 0.0063 | 0.0794 |  |
| **Deficiency Anemia** | | |  |  |  |  |  |  |  |  |  |
|  | No | | 0.3019 | 0.7055 | 0.3973 | 0.0039 | 0.0752 | 0.0579 | 0.0061 | 0.0971 | 0.0392 |
|  | Yes | | 0.0800 | 0.3923 |  | 0.0007 | 0.0257 |  | 0.0044 | 0.0695 |  |
| **Alcohol Abuse** | | |  |  |  |  |  |  |  |  |  |
|  | No | | 0.3017 | 0.7054 | 0.3061 | 0.0039 | 0.0751 | 0.0625 | 0.0061 | 0.0971 | 0.0265 |
|  | Yes | | 0.0512 | 0.3187 |  | 0.0022 | 0.0464 |  | 0.0018 | 0.0496 |  |
| **Drug Abuse** | | |  |  |  |  |  |  |  |  |  |
|  | No | | 0.3009 | 0.7046 | 0.785 | 0.0038 | 0.0750 | 0.6711 | 0.0060 | 0.0970 | 0.6608 |
|  | Yes | | 0.0000 | 0.0000 |  | 0.0000 | 0.0000 |  | 0.0000 | 0.0000 |  |
| **Psychoses** | | |  |  |  |  |  |  |  |  |  |
|  | No | | 0.3017 | 0.7053 | 0.1161 | 0.0039 | 0.0751 | <.0001 | 0.0061 | 0.0971 | <.0001 |
|  | Yes | | 0.0406 | 0.3305 |  | 0.0003 | 0.0179 |  | 0.0010 | 0.0311 |  |
| **Depression** | | |  |  |  |  |  |  |  |  |  |
|  | No | | 0.3028 | 0.7063 | 0.3571 | 0.0038 | 0.0750 | <.0001 | 0.0059 | 0.0956 | <.0001 |
|  | Yes | | 0.0871 | 0.4353 |  | 0.0039 | 0.0788 |  | 0.0249 | 0.1972 |  |
| **Total** |  | | 0.2742 | 0.7321 |  | 0.0045 | 0.0870 |  | 0.0078 | 0.1171 |  |

| **Table S3. Interrupted time series analysis of the effect of the over-the-counter drug policy on number of monthly outpatient visits for acute upper respiratory infections, dyspepsia, and migraine (Full model)** | | | | | | | | | | | | | | | | | | | | | | | |
| --- | --- | --- | --- | --- | --- | --- | --- | --- | --- | --- | --- | --- | --- | --- | --- | --- | --- | --- | --- | --- | --- | --- | --- |
|  |  | **Acute upper respiratory infections** | | | | | | **Dyspepsia** | | | | | | | | **Migraine** | | | | | | | |
|  |  | **Estimation** | | **Standard error** | | **p-value** | | **Estimation** | | | **Standard error** | | | **p-value** | | **Estimation** | | | | **Standard error** | | | **p-value** |
| **Trend** |  | -0.0003 | 0.0001 | | <.0001 | | 0.0101 | | | 0.0005 | | | <.0001 | | 0.0057 | | | | 0.0003 | | | <.0001 | |
| **Intervention** | |  |  | |  | |  | | |  | | |  | |  | | | |  | | |  | |
|  | Yes | 0.0026 | 0.0017 | | 0.1307 | | -0.0216 | | | 0.0194 | | | 0.2651 | | -0.0314 | | | | 0.0151 | | | 0.037 | |
|  | No | Ref. | - | | - | | Ref. | | | - | | | - | | Ref. | | | | - | | | - | |
| **Trend after intervention** | | 0.0002 | 0.0002 | | 0.3416 | | -0.007 | | | 0.0021 | | | 0.0007 | | 0.0019 | | | | 0.0016 | | | 0.2497 | |
| **Sex** |  |  |  | |  | |  | | |  | | |  | |  | | | |  | | |  | |
|  | Male | -0.0137 | 0.0015 | | <.0001 | | -0.2174 | | | 0.0144 | | | <.0001 | | -0.4796 | | | | 0.0149 | | | <.0001 | |
|  | Female | Ref. | - | | - | | Ref. | | | - | | | - | | Ref. | | | | - | | | - | |
| **Age** |  |  |  | |  | |  | | |  | | |  | |  | | | |  | | |  | |
|  | less than 30 | 0.568 | 0.0107 | | <.0001 | | 1.2492 | | | 0.0406 | | | <.0001 | | 1.0934 | | | | 0.0429 | | | <.0001 | |
|  | 30-39 | 0.4174 | 0.0108 | | <.0001 | | 1.1252 | | | 0.0422 | | | <.0001 | | 1.4585 | | | | 0.0426 | | | <.0001 | |
|  | 40-49 | 0.3975 | 0.0108 | | <.0001 | | 0.8341 | | | 0.0426 | | | <.0001 | | 1.344 | | | | 0.0427 | | | <.0001 | |
|  | 50-59 | 0.3842 | 0.0109 | | <.0001 | | 0.462 | | | 0.0433 | | | <.0001 | | 1.0162 | | | | 0.0429 | | | <.0001 | |
|  | 60-69 | 0.3177 | 0.0113 | | <.0001 | | 0.0865 | | | 0.0438 | | | 0.0484 | | 0.5039 | | | | 0.0441 | | | <.0001 | |
|  | 70-79 | 0.1888 | 0.0115 | | <.0001 | | -0.0287 | | | 0.0448 | | | 0.5216 | | 0.1723 | | | | 0.0451 | | | 0.0001 | |
|  | 80 or more | Ref. | - | | - | | Ref. | | | - | | | - | | Ref. | | | | - | | | - | |
| **Insurance** |  |  |  | |  | |  | | |  | | |  | |  | | | |  | | |  | |
|  | Self-employed insured | Ref. | - | | - | | Ref. | | | - | | | - | | Ref. | | | | - | | | - | |
|  | employee insured | 0.0004 | 0.0062 | | 0.9423 | | 0.0868 | | | 0.0424 | | | 0.0406 | | 0.1812 | | | | 0.0406 | | | <.0001 | |
|  | Medicaid | 0.0215 | 0.0063 | | 0.0006 | | 0.1346 | | | 0.0438 | | | 0.0021 | | 0.1915 | | | | 0.042 | | | <.0001 | |
| **Income** |  |  |  | |  | |  | | |  | | |  | |  | | | |  | | |  | |
|  | Q1(Low) | -0.0459 | 0.0024 | | <.0001 | | -0.2681 | | | 0.0225 | | | <.0001 | | -0.1883 | | | | 0.0198 | | | <.0001 | |
|  | Q2 | -0.0407 | 0.002 | | <.0001 | | -0.2768 | | | 0.0183 | | | <.0001 | | -0.2068 | | | | 0.017 | | | <.0001 | |
|  | Q3 | 0.0047 | 0.0016 | | 0.0043 | | -0.185 | | | 0.0167 | | | <.0001 | | -0.1625 | | | | 0.0154 | | | <.0001 | |
|  | Q4(High) | Ref. | - | | - | | Ref. | | | - | | | - | | Ref. | | | | - | | | - | |
| **Region** |  |  |  | |  | |  | | |  | | |  | |  | | | |  | | |  | |
|  | Urban | 0.0646 | 0.0015 | | <.0001 | | 0.5416 | | | 0.0137 | | | <.0001 | | 0.5124 | | | | 0.013 | | | <.0001 | |
|  | Rural | Ref. | - | | - | | Ref. | | | - | | | - | | Ref. | | | | - | | | - | |
| **Type of hospital** | |  |  | |  | |  | | |  | | |  | |  | | | |  | | |  | |
|  | General hospital | -0.0509 | 0.0071 | | <.0001 | | -0.0013 | | | 0.0385 | | | <.0001 | | -0.0149 | | | | 0.0275 | | | <.0001 | |
|  | Hospital | -0.0132 | 0.0047 | | <.0001 | | -0.0062 | | | 0.0284 | | | <.0001 | | -0.058 | | | | 0.0208 | | | <.0001 | |
|  | Clinic | -0.002 | 0.0044 | | <.0001 | | 0.1791 | | | 0.0245 | | | <.0001 | | -0.0505 | | | | 0.0184 | | | <.0001 | |
|  | Others | Ref. | - | | - | | Ref. | | | - | | | - | | Ref. | | | | - | | | - | |
| **Admission in last month** | |  |  | |  | |  | | |  | | |  | |  | | | |  | | |  | |
|  | Yes | Ref. | - | | - | | Ref. | | | - | | | - | | Ref. | | | | - | | | - | |
|  | No | 0.0262 | 0.0031 | | <.0001 | | -0.2025 | | | 0.0257 | | | <.0001 | | -0.1626 | | | | 0.0197 | | | <.0001 | |
| **Season** |  |  |  | |  | |  | | |  | | |  | |  | | | |  | | |  | |
|  | Spring | 0.0113 | 0.0009 | | <.0001 | | 0.0046 | | | 0.0102 | | | 0.654 | | 0.0762 | | | | 0.0075 | | | <.0001 | |
|  | Summer | -0.0801 | 0.0011 | | <.0001 | | 0.0147 | | | 0.011 | | | 0.181 | | 0.136 | | | | 0.008 | | | <.0001 | |
|  | Fall | -0.0065 | 0.0009 | | <.0001 | | -0.0495 | | | 0.0106 | | | <.0001 | | 0.0512 | | | | 0.0076 | | | <.0001 | |
|  | Winter | Ref. | - | | - | | Ref. | | | - | | | - | | Ref. | | | | - | | | - | |
| **Congestive Heart Failure** | |  |  | |  | |  | | |  | | |  | |  | | | |  | | |  | |
|  | No | -0.3155 | 0.0244 | | <.0001 | | -1.2311 | | | 0.1163 | | | <.0001 | | -0.6864 | | | | 0.0835 | | | <.0001 | |
|  | Yes | Ref. | - | | - | | Ref. | | | - | | | - | | Ref. | | | | - | | | - | |
| **Cardiac Arrhythmia** | |  |  | |  | |  | | |  | | |  | |  | | | |  | | |  | |
|  | No | -0.2632 | 0.0222 | | <.0001 | | -0.9392 | | | 0.1217 | | | <.0001 | | -0.8535 | | | | 0.0742 | | | <.0001 | |
|  | Yes | Ref. | - | | - | | Ref. | | | - | | | - | | Ref. | | | | - | | | - | |
| **Valvular Disease** | |  |  | |  | |  | | |  | | |  | |  | | | |  | | |  | |
|  | No | -0.2274 | 0.0399 | | <.0001 | | -1.1749 | | | 0.4136 | | | 0.0045 | | -0.1981 | | | | 0.1987 | | | 0.3188 | |
|  | Yes | Ref. | - | | - | | Ref. | | | - | | | - | | Ref. | | | | - | | | - | |
| **Pulmonary Circulation Disorders** | | | | |  | | | |  | | |  | | | | |  | | | |  | | |
|  | No | -0.0444 | 0.0709 | | 0.531 | | 0.1268 | | | 0.3044 | | | 0.6771 | | 0.0521 | | | | 0.3221 | | | 0.8714 | |
|  | Yes | Ref. | - | | - | | Ref. | | | - | | | - | | Ref. | | | | - | | | - | |
| **Peripheral Vascular Disorders** | |  |  | |  | |  | | |  | | |  | |  | | | |  | | |  | |
|  | No | -0.0058 | 0.0141 | | 0.6803 | | -0.1202 | | | 0.0636 | | | 0.0587 | | -0.3939 | | | | 0.0353 | | | <.0001 | |
|  | Yes | Ref. | - | | - | | Ref. | | | - | | | - | | Ref. | | | | - | | | - | |
| **Hypertension Uncomplicated** | |  |  | |  | |  | | |  | | |  | |  | | | |  | | |  | |
|  | No | 0.9264 | 0.0074 | | <.0001 | | 1.8376 | | | 0.0304 | | | <.0001 | | 1.4638 | | | | 0.0224 | | | <.0001 | |
|  | Yes | Ref. | - | | - | | Ref. | | | - | | | - | | Ref. | | | | - | | | - | |
| **Hypertension Complicated** | |  |  | |  | |  | | |  | | |  | |  | | | |  | | |  | |
|  | No | 0.2908 | 0.0236 | | <.0001 | | 0.8552 | | | 0.1047 | | | <.0001 | | 0.3776 | | | | 0.069 | | | <.0001 | |
|  | Yes | Ref. | - | | - | | Ref. | | | - | | | - | | Ref. | | | | - | | | - | |
| **Paralysis** |  |  |  | |  | |  | | |  | | |  | |  | | | |  | | |  | |
|  | No | 0.5093 | 0.0554 | | <.0001 | | 0.0852 | | | 0.2115 | | | 0.6872 | | 0.3839 | | | | 0.1548 | | | 0.0131 | |
|  | Yes | Ref. | - | | - | | Ref. | | | - | | | - | | Ref. | | | | - | | | - | |
| **Other Neurological Disorders** | |  |  | |  | |  | | |  | | |  | |  | | | |  | | |  | |
|  | No | -0.0047 | 0.0168 | | 0.781 | | -0.2772 | | | 0.1203 | | | 0.0213 | | -0.4549 | | | | 0.0491 | | | <.0001 | |
|  | Yes | Ref. | - | | - | | Ref. | | | - | | | - | | Ref. | | | | - | | | - | |
| **Chronic Pulmonary Disease** | |  |  | |  | |  | | |  | | |  | |  | | | |  | | |  | |
|  | No | -0.1481 | 0.002 | | <.0001 | | -0.2364 | | | 0.0359 | | | <.0001 | | 0.0051 | | | | 0.0336 | | | 0.8804 | |
|  | Yes | Ref. | - | | - | | Ref. | | | - | | | - | | Ref. | | | | - | | | - | |
| **Diabetes Uncomplicated** | |  |  | |  | |  | | |  | | |  | |  | | | |  | | |  | |
|  | No | 0.265 | 0.0148 | | <.0001 | | 0.7451 | | | 0.0497 | | | <.0001 | | 0.7057 | | | | 0.0455 | | | <.0001 | |
|  | Yes | Ref. | - | | - | | Ref. | | | - | | | - | | Ref. | | | | - | | | - | |
| **Diabetes Complicated** | |  |  | |  | |  | | |  | | |  | |  | | | |  | | |  | |
|  | No | 0.0155 | 0.0166 | | 0.3495 | | 0.2787 | | | 0.0701 | | | <.0001 | | 0.2707 | | | | 0.0719 | | | 0.0002 | |
|  | Yes | Ref. | - | | - | | Ref. | | | - | | | - | | Ref. | | | | - | | | - | |
| **Hypothyroidism** | |  |  | |  | |  | | |  | | |  | |  | | | |  | | |  | |
|  | No | -0.1289 | 0.0143 | | <.0001 | | -0.6698 | | | 0.1186 | | | <.0001 | | -0.3629 | | | | 0.0893 | | | <.0001 | |
|  | Yes | Ref. | - | | - | | Ref. | | | - | | | - | | Ref. | | | | - | | | - | |
| **Renal Failure** | |  |  | |  | |  | | |  | | |  | |  | | | |  | | |  | |
|  | No | -0.0201 | 0.0395 | | 0.6112 | | -0.8846 | | | 0.1514 | | | <.0001 | | -0.3794 | | | | 0.1508 | | | 0.0119 | |
|  | Yes | Ref. | - | | - | | Ref. | | | - | | | - | | Ref. | | | | - | | | - | |
| **Liver Disease** | |  |  | |  | |  | | |  | | |  | |  | | | |  | | |  | |
|  | No | -0.1071 | 0.0075 | | <.0001 | | -0.7115 | | | 0.0407 | | | <.0001 | | -0.5373 | | | | 0.0333 | | | <.0001 | |
|  | Yes | Ref. | - | | - | | Ref. | | | - | | | - | | Ref. | | | | - | | | - | |
| **Peptic Ulcer Disease excluding bleeding** | | | | | |  | | | | | |  | | | | | |  | | | | | |
|  | No | -0.0838 | 0.0041 | | <.0001 | | -1.1228 | | | 0.0348 | | | <.0001 | | -0.7558 | | | | 0.0255 | | | <.0001 | |
|  | Yes | Ref. | - | | - | | Ref. | | | - | | | - | | Ref. | | | | - | | | - | |
| **AIDS/HIV** | |  |  | |  | |  | | |  | | |  | |  | | | |  | | |  | |
|  | No | 0.1521 | 0.1353 | | 0.2607 | | -0.513 | | | 0.6085 | | | 0.3991 | | -8.6731 | | | | 0.0958 | | | <.0001 | |
|  | Yes | Ref. | - | | - | | Ref. | | | - | | | - | | Ref. | | | | - | | | - | |
| **Lymphoma** | |  |  | |  | |  | | |  | | |  | |  | | | |  | | |  | |
|  | No | 0.054 | 0.0995 | | 0.5874 | | -1.0442 | | | 0.5094 | | | 0.0404 | | 1.2847 | | | | 0.7079 | | | 0.0696 | |
|  | Yes | Ref. | - | | - | | Ref. | | | - | | | - | | Ref. | | | | - | | | - | |
| **Metastatic Cancer** | |  |  | |  | |  | | |  | | |  | |  | | | |  | | |  | |
|  | No | 0.1214 | 0.0497 | | 0.0145 | | 0.057 | | | 0.3449 | | | 0.8688 | | 0.2609 | | | | 0.2023 | | | 0.1972 | |
|  | Yes | Ref. | - | | - | | Ref. | | | - | | | - | | Ref. | | | | - | | | - | |
| **Solid Tumor without Metastasis** | |  |  | |  | |  | | |  | | |  | |  | | | |  | | |  | |
|  | No | 0.0739 | 0.0143 | | <.0001 | | -0.524 | | | 0.0664 | | | <.0001 | | 0.1284 | | | | 0.0782 | | | 0.1005 | |
|  | Yes | Ref. | - | | - | | Ref. | | | - | | | - | | Ref. | | | | - | | | - | |
| **Rheumatoid Arthritis/collagen** | |  |  | |  | |  | | |  | | |  | |  | | | |  | | |  | |
|  | No | -0.0122 | 0.0152 | | 0.4218 | | -0.4531 | | | 0.1756 | | | 0.0099 | | -0.2401 | | | | 0.0716 | | | 0.0008 | |
|  | Yes | Ref. | - | | - | | Ref. | | | - | | | - | | Ref. | | | | - | | | - | |
| **Coagulopathy** | |  |  | |  | |  | | |  | | |  | |  | | | |  | | |  | |
|  | No | 0.1758 | 0.0472 | | 0.0002 | | 0.5258 | | | 0.2292 | | | 0.0218 | | 0.5369 | | | | 0.1679 | | | 0.0014 | |
|  | Yes | Ref. | - | | - | | Ref. | | | - | | | - | | Ref. | | | | - | | | - | |
| **Obesity** |  |  |  | |  | |  | | |  | | |  | |  | | | |  | | |  | |
|  | No | 0.2076 | 0.0435 | | <.0001 | | 0.3585 | | | 0.1848 | | | 0.0524 | | 0.7194 | | | | 0.2404 | | | 0.0028 | |
|  | Yes | Ref. | - | | - | | Ref. | | | - | | | - | | Ref. | | | | - | | | - | |
| **Weight Loss** | |  |  | |  | |  | | |  | | |  | |  | | | |  | | |  | |
|  | No | 0.1036 | 0.0465 | | 0.0257 | | -0.2852 | | | 0.1508 | | | 0.0585 | | 0.1297 | | | | 0.1559 | | | 0.4055 | |
|  | Yes | Ref. | - | | - | | Ref. | | | - | | | - | | Ref. | | | | - | | | - | |
| **Fluid and Electrolyte Disorders** | |  |  | |  | |  | | |  | | |  | |  | | | |  | | |  | |
|  | No | -0.0792 | 0.0083 | | <.0001 | | 0.0155 | | | 0.0868 | | | 0.8586 | | -0.2129 | | | | 0.0557 | | | 0.0001 | |
|  | Yes | Ref. | - | | - | | Ref. | | | - | | | - | | Ref. | | | | - | | | - | |
| **Blood Loss Anemia** | |  |  | |  | |  | | |  | | |  | |  | | | |  | | |  | |
|  | No | 0.0074 | 0.0539 | | 0.8911 | | -0.2969 | | | 0.2465 | | | 0.2284 | | -0.0511 | | | | 0.2039 | | | 0.8021 | |
|  | Yes | Ref. | - | | - | | Ref. | | | - | | | - | | Ref. | | | | - | | | - | |
| **Deficiency Anemia** | |  |  | |  | |  | | |  | | |  | |  | | | |  | | |  | |
|  | No | -0.049 | 0.0108 | | <.0001 | | -0.2104 | | | 0.0899 | | | 0.0193 | | -0.2225 | | | | 0.0506 | | | <.0001 | |
|  | Yes | Ref. | - | | - | | Ref. | | | - | | | - | | Ref. | | | | - | | | - | |
| **Alcohol Abuse** | |  |  | |  | |  | | |  | | |  | |  | | | |  | | |  | |
|  | No | 0.2314 | 0.028 | | <.0001 | | 0.2985 | | | 0.1132 | | | 0.0084 | | 0.2566 | | | | 0.0839 | | | 0.0022 | |
|  | Yes | Ref. | - | | - | | Ref. | | | - | | | - | | Ref. | | | | - | | | - | |
| **Drug Abuse** | |  |  | |  | |  | | |  | | |  | |  | | | |  | | |  | |
|  | No | 0.2298 | 0.2114 | | 0.277 | | -0.5132 | | | 0.2342 | | | 0.0284 | | 0.4614 | | | | 0.2282 | | | 0.0432 | |
|  | Yes | Ref. | - | | - | | Ref. | | | - | | | - | | Ref. | | | | - | | | - | |
| **Psychoses** | |  |  | |  | |  | | |  | | |  | |  | | | |  | | |  | |
|  | No | 0.3426 | 0.0302 | | <.0001 | | 0.1653 | | | 0.1668 | | | 0.3216 | | 0.5987 | | | | 0.1376 | | | <.0001 | |
|  | Yes | Ref. | - | | - | | Ref. | | | - | | | - | | Ref. | | | | - | | | - | |
| **Depression** | |  |  | |  | |  | | |  | | |  | |  | | | |  | | |  | |
|  | No | -0.1365 | 0.0096 | | <.0001 | | -1.1457 | | | 0.058 | | | <.0001 | | -1.0106 | | | | 0.0228 | | | <.0001 | |
|  | Yes | Ref. | - | | - | | Ref. | | | - | | | - | | Ref. | | | | - | | | - | |
